# Human Umbilical Cord Mesenchymal Stem Cell‐Derived Exosomes Attenuate IMQ‐Induced Psoriasiform Dermatitis: A Preclinical Experimental Study of Exosomal miR‐125b‐5p/STAT3 Signaling

**DOI:** 10.1155/sci/4935728

**Published:** 2026-09-25

**Authors:** Ziyan Li, Rui Ren, Yunjia Wang, Zhichao Fan, Yijun Zhang, Jieling Fu, Jiaxin Huang, Yuting Huang, Kaiyuan Zhang, Qian Li

**Affiliations:** ^1^ School of Life Science and Technology, China Pharmaceutical University, Nanjing 211198, China, cpu.edu.cn; ^2^ School of Traditional Chinese Pharmacy, China Pharmaceutical University, Nanjing 211198, China, cpu.edu.cn

**Keywords:** exosome, mesenchymal stem cell, miR-125b-5p, psoriasis

## Abstract

**Background and Aims:**

Psoriasis is a chronic inflammatory skin disease driven largely by dysregulation of the IL‐23/Th17 axis and excessive keratinocyte proliferation. Human umbilical cord mesenchymal stem cell (MSC)‐derived exosomes (hucMSC‐Exos) represent a potential cell‐free therapeutic strategy, but their mechanisms remain incompletely defined. This study evaluated the effects of hucMSC‐Exos in psoriasiform inflammation and investigated the contribution of exosomal miR‐125b‐5p and its relationship with STAT3 signaling.

**Methods:**

hucMSC‐Exos were isolated by differential ultracentrifugation and characterized by nanoparticle tracking analysis (NTA), transmission electron microscopy (TEM), and immunoblotting. Therapeutic effects were assessed in an imiquimod (IMQ)‐induced mouse model of psoriasiform dermatitis using PASI‐like scoring, histopathology, immunohistochemistry, flow cytometry, and western blotting. Exosomal microRNAs (miRNAs) were profiled by high‐throughput sequencing. STAT3 targeting was examined by dual‐luciferase reporter assays, and the functional contribution of miR‐125b‐5p was assessed using exosomes loaded with a miR‐125b‐5p inhibitory oligonucleotide and negative‐control exosomes (NC‐Exos).

**Results:**

hucMSC‐Exos alleviated IMQ‐induced psoriasiform dermatitis, as reflected by reduced epidermal hyperplasia and fewer Ki‐67‐positive keratinocytes. Exosome treatment was associated with decreased phosphorylation of STAT3 and JNK, lower IL‐23 and IL‐17A expression, decreased Th17‐cell frequency, and increased regulatory T cell (Treg)‐cell frequency. In vitro, hucMSC‐Exos suppressed Th17‐cell proliferation while promoting Treg‐cell expansion. miR‐125b‐5p was among the abundant miRNAs detected in hucMSC‐Exos, and dual‐luciferase reporter assays supported STAT3 as a direct target of miR‐125b‐5p. Compared with NC‐Exos, inhibitor‐loaded exosomes (IN‐Exos) were less effective at alleviating IMQ‐induced psoriasiform dermatitis; this reduced efficacy was accompanied by increased STAT3 and JNK phosphorylation, higher IL‐23 and IL‐17A expression, a higher Th17‐cell frequency, and a lower Treg‐cell frequency.

**Conclusion:**

hucMSC‐Exos attenuated psoriasiform inflammation and were associated with reduced inflammatory signaling, decreased Th17‐cell frequency, and increased Treg‐cell frequency. Our findings further suggest that exosomal miR‐125b‐5p contributes, at least in part, to these effects through direct regulation of STAT3.

## 1. Introduction

Psoriasis, a chronic immune‐mediated dermatosis, affects ~2%–3% of the population worldwide [1]. Characterized by aberrant keratinocyte proliferation and differentiation, neoangiogenesis, and leukocyte infiltration [2], it arises from complex gene‐environment interactions [3]. While HLA‐C
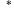
06:02 polymorphisms confer genetic susceptibility [4], environmental triggers including mechanical trauma, microbial exposure, pharmacological agents, and metabolic dysregulation initiate disease flares [5, 6]. Importantly, the immunopathogenesis centers on IL‐23/Th17 axis dysregulation [7], wherein dendritic cell‐derived IL‐23 promotes Th17 differentiation, driving IL‐17/TNF‐α‐mediated keratinocyte hyperactivation through autocrine or paracrine loops [8, 9]. Current biologics targeting this axis (anti‐IL‐23/IL‐17) and TNF‐α inhibitors demonstrate clinical efficacy [10, 11], such as ustekinumab, guselkumab, secukinumab, ixekizumab, and adalimumab, yet persistent therapeutic gaps remain regarding long‐term safety and refractoriness.

Numerous studies indicate that mesenchymal stem cells (MSCs) exhibit multilineage differentiation capacity and immunomodulatory properties through paracrine mechanisms [12, 13]. Recently, emerging evidence suggests that cell‐derived extracellular vesicles (EVs) transfer bioactive molecules to regulate the development of various inflammatory diseases [14, 15]. Previous studies have suggested that MSC‐derived exosomes may exert anti‐psoriatic effects through multiple mechanisms. For example, upregulation of TGF‐β2 has been proposed as one mechanism by which MSC‐derived exosomes reduce psoriatic hyperplasia [16], whereas human umbilical cord MSC‐derived exosomes (hucMSC‐Exos) have been shown to alleviate psoriasis‐like inflammation through modulation of the IL‐23/IL‐17 axis, STAT3 signaling, and dendritic cell activity [17]. These findings indicate that the therapeutic effects of MSC‐derived exosomes are likely mediated by multiple bioactive components and signaling pathways. However, the contribution of specific exosomal microRNAs (miRNAs) to these immunomodulatory effects remains incompletely understood. Therefore, the present study investigated whether exosomal miR‐125b‐5p contributes to the therapeutic effects of hucMSC‐Exos through the regulation of STAT3 signaling.

Exosomes are 30–200 nm lipid‐bilayer vesicles that contain bioactive molecules, including miRNAs, proteins, and metabolites. miRNAs are a class of ~20–22‐nucleotide noncoding RNAs that negatively regulate target gene expression at the posttranscriptional level, primarily through complementary binding to sequences within the 3′ untranslated region (3′ UTR) of target mRNAs. miRNAs are involved in the regulation of chronic inflammatory and immune processes [18]. In recent years, miRNAs carried by MSC‐derived exosomes have been investigated for their therapeutic potential in immune and inflammatory diseases, including colitis [19], rheumatoid arthritis [20], and autoimmune hepatitis [21]. Exosomal miRNA cargo can contribute to the posttranscriptional regulation of genes involved in inflammatory and immune responses [22–24]. However, the specific miRNA components and molecular targets responsible for the therapeutic effects of hucMSC‐Exos in psoriasis remain incompletely understood.

The JNK signaling cascade, a MAP kinase subfamily governing cellular stress responses, exhibits pathological activation in the psoriatic epidermis. Damage‐associated molecular patterns (DAMPs) activate JNK‐mediated transcriptional programs, upregulating proinflammatory mediators (IL‐6, IL‐8, IL‐23, CCL20, hβD‐2) that sustain keratinocyte hyperproliferation and immune cell recruitment [25–28]. Concurrently, STAT3 hyperphosphorylation in lesional keratinocytes induces proliferative and anti‐apoptotic gene signatures [29, 30], as evidenced by psoriasis‐form phenotypes in K5.STAT3C transgenic models [31, 32]. JNK and STAT3 are both involved in inflammatory signaling, and previous studies in other disease contexts have suggested that JNK can contribute to STAT3 activation. However, the relationship between these pathways is context‐dependent, and their functional hierarchy in psoriasis remains incompletely defined.

This study investigates the therapeutic mechanisms of hucMSC‐Exos in imiquimod (IMQ)‐induced psoriasiform dermatitis. Through integrated miRNA sequencing and functional validation, we investigated the contribution of exosomal miR‐125b‐5p to the therapeutic effects of hucMSC‐Exos and examined its relationship with STAT3‐associated inflammatory signaling. Our findings provide mechanistic justification for the clinical translation of hucMSC‐Exos as a novel biologic strategy for psoriasis management.

## 2. Materials and Methods

### 2.1. Isolation and Characterization of Human Umbilical Cord MSCs (hucMSCs)

hucMSCs were obtained from Jiangsu Puzhongkang Biotechnology Co., Ltd. (Taizhou, Jiangsu, China). According to the supplier, the hucMSCs were isolated from umbilical cords voluntarily donated by healthy women after full‐term delivery. Written informed consent for the donation and use of the umbilical cord tissues for research purposes was obtained from all donors prior to sample collection. Cells were cultured according to the manufacturer’s instructions and maintained at 37°C in a humidified 5% CO_2_ atmosphere using α‐MEM basal medium (KenGEN BioTECH, China) supplemented with 10% fetal bovine serum (Gibco, USA) and 1% penicillin‐streptomycin (100 U·mL^−1^ penicillin, 100 μg·mL^−1^ streptomycin; Beyotime, China).

Multilineage differentiation potential was validated through lineage‐specific induction assays: osteogenic differentiation was assessed by alizarin red S staining (OriCell, China) after 15 days of culture in osteogenic induction medium (OriCell, China); adipogenesis was evaluated via oil red O staining (OriCell, China) following 14 days of adipogenic induction medium (OriCell, China); and chondrogenic differentiation was confirmed by Alcian Blue staining (OriCell, China) using a pellet culture system with chondrogenic medium (OriCell, China) for 20 days.

Flow cytometric immunophenotyping was performed using FITC‐conjugated monoclonal antibodies human anti‐CD44, anti‐CD45, anti‐CD73, and anti‐CD19 (Proteintech, China) with FITC‐IgG1 (Proteintech, China) isotype controls. Cell suspensions (1 × 10^6^ cells/mL) were analyzed on a FACSCelesta flow cytometer (BD Biosciences), with data processed using FlowJo v10.8.1 (BD biosciences, USA).

### 2.2. Isolation and Characterization of hucMSC‐Exos

hucMSCs at passages 3–6 were cultured in α‐MEM supplemented with 2% exosome‐depleted fetal bovine serum (BDBIO, Zhejiang, China). The conditioned medium was collected after 48 h for exosome isolation. This passage range reflects the cells used in the present study and was not intended to define a validated upper passage limit for exosome quality.

Exosomes were purified from conditioned media through differential ultracentrifugation: sequential centrifugation at 300 × *g* (10 min), 2000 × *g* (20 min), and 10,000 × *g* (40 min) removed cellular debris, followed by 0.22 μm filtration (BIOFIL, China) and ultracentrifugation at 120,000 × *g* (70 min, 4°C; Optima XE‐100, Beckman Coulter). Pellets were washed in PBS and ultracentrifuged at 120,000 × *g* (70 min, 4°C) again prior to resuspension in PBS (100 μL) and storage at −80°C.

Nanoparticle tracking analysis (NTA) was conducted using a NanoSight NS300 system (Malvern Panalytical). Transmission electron microscopy (TEM) samples were prepared by negative staining with 2% uranyl acetate and imaged at 80 kV (HT7800, Hitachi). Western blot confirmed exosomal markers (CD9, CD81, CD63, Alix, and TSG101) and the absence of calnexin (ER marker).

### 2.3. Preparation of miR‐125b‐5p Inhibitor‐Loaded Exosomes (IN‐Exos)

Purified hucMSC‐Exos were loaded with a miR‐125b‐5p inhibitor or a negative control oligonucleotide using the Exo‐Fect Exosome Transfection Reagent (System Biosciences, USA) according to the manufacturer’s instructions. Briefly, 50 μL of purified exosomes was mixed with 10 μL of Exo‐Fect solution, 20 pmol of miR‐125b‐5p inhibitor or negative control oligonucleotide, and 70 μL of sterile 1× PBS. The reaction mixture was incubated at 37°C for 10 min. Subsequently, 30 μL of ExoQuick‐TC was added, followed by incubation on ice for 30 min. The samples were centrifuged at 13,000–14,000 rpm for 3 min, and the resulting exosome pellets were resuspended in 300 μL of sterile 1 × PBS. Exosomes loaded with the miR‐125b‐5p inhibitor were designated as IN‐Exos, whereas exosomes loaded with the negative control inhibitor were designated as negative‐control exosomes (NC‐Exos).

### 2.4. Western Blot Assay

Total cellular protein and exosomal protein were extracted using radioimmunoprecipitation assay (RIPA) lysis buffer containing a protease inhibitor cocktail (epizyme, China). Protein extracts were separated by 10% sodium dodecyl sulfate‐polyacrylamide gel electrophoresis (SDS‐PAGE) and transferred onto polyvinylidene fluoride (PVDF) membranes (Merck Millipore, USA). After blocking for 2 h, membranes were incubated with primary antibodies overnight at 4 °C and then with HRP‐conjugated secondary antibodies for 2 h. Afterward, membranes were incubated with the Immobilon ECL substrate kit (YEASEN, China) for 1 min and detected by the Tanon 4600 Imaging System (Tanon, China). Protein band intensities were analyzed with ImageJ software. The primary antibodies used in the experiments included IL‐17A (SantaCruz, sc‐374218, 1:1000), IL‐23 (SantaCruz, sc‐279, 1:1000), Stat3 (Cell Signaling, 124H6, 1:1000), Phospho‐Stat3 (Tyr705) (Cell Signaling, D3A7, 1:2000), JNK (D‐2) (SantaCruz, sc‐7345, 1:1000), Phospho‐JNK (G‐7) (SantaCruz, SC‐6254, 1:1000), HSP70 (Proteintech, 10995‐1‐AP, 1:5000), CD9 (Cell Signaling, D8O1A,1:1000), Alix (Proteintech, 12422‐1‐AP, 1:2000), calnexin (HUABIO, SN20‐54, 1:1000), CD81 (HUABIO, SN206‐01, 1:1000), CD63 (HUABIO, SY21‐02, 1:1000), and β‐Actin (HUABIO, PSH03‐63, 1:20,000). The secondary antibodies used in the experiments included Goat anti‐rabbit IgG‐HRP antibody (HUABIO, 1:50,000) and Goat anti‐mouse IgG‐HRP antibody (HUABIO, 1:20,000).

### 2.5. Psoriasiform Dermatitis Model and Therapeutic Intervention

All animal procedures were conducted in accordance with the institutional guidelines for the care and use of laboratory animals and were approved by the Ethics Committee of China Pharmaceutical University (Approval Number YSL‐202508025) Male BALB/c mice (6–8 weeks; SPF grade; Jiangsu Huachuang SinoPharmTech Co.) received daily topical application of 62.5 mg IMQ cream (5% IMQ; Sichuan Mingxin) on depilated dorsal skin for six consecutive days. hucMSC‐Exos (100 μg in 200 μL PBS) were administered subcutaneously on days 1, 3, and 5. Disease severity was evaluated using a modified PASI‐like scoring system. Erythema, scaling, and skin thickening were independently scored on a scale from 0 to 4 (0, none; 1, slight; 2, moderate; 3, marked; and 4, very marked). The composite PASI‐like score was calculated as the sum of the erythema, scaling, and thickening scores, yielding a total score ranging from 0 to 12. The cumulative score (sum of erythema, scaling, and thickening scores) was used to indicate the severity of inflammation on a scale of 0–12.

### 2.6. CD4^+^ T Cell Isolation, Th17 Polarization, and Regulatory T Cell (Treg) Polarization

Splenic CD4^+^ T cells were isolated via magnetic‐activated cell sorting (MojoSort Mouse CD4^+^ T Cell Isolation Kit, BioLegend, USA). Cells were activated with plate‐bound anti‐CD3ε/anti‐CD28 (10 μg·mL^−1^ each; BioLegend, USA) under Th17‐polarizing conditions: IL‐1β (10 ng·mL^−1^), IL‐6 (10 ng·mL^−1^), IL‐23 (10 ng·mL^−1^), TGF‐β (2.5 ng·mL^−1^), anti‐IFN‐γ (10 μg·mL^−1^), and anti‐IL‐4 (10 μg·mL^−1^; PeproTech, USA). Cells were activated with plate‐bound anti‐CD3ε/anti‐CD28 (10 μg·mL^−1^ each; BioLegend, USA) under Treg‐polarizing conditions: TGF‐β (5 ng·mL^−1^) and IL‐2 (10 ng·mL^−1^; PeproTech, USA).

### 2.7. Flow Cytometry

For intracellular cytokine staining, cells were stimulated with Brefeldin A Solution (1000X) (Biolegend, USA) and Monensin Solution (1000X) (Biolegend, USA) for 4 h. After staining for surface markers, cells were permeabilized with Intracellular Staining Permeabilization Wash Buffer (Biolegend, USA) and intracellularly stained for corresponding antibodies. The labeled antibodies include FITC anti‐mouse CD4 (Biolegend, USA), PE/Cyanine7 anti‐mouse IL‐17 (Biolegend, USA), Brilliant Violet 421 anti‐mouse Foxp3 (Biolegend, USA), and APC anti‐mouse CD25 (Biolegend, USA). Then, cells were analyzed by flow cytometry (FACS Celesta system, BD Biosciences, USA) and analyzed by FlowJo software.

### 2.8. Carboxyfluorescein Succinimidyl Ester (CFSE) Dilution Assay

The CFSE dilution assay was performed using CellTrace CFSE Cell Proliferation Kit (Thermo Fisher Scientific Inc, USA) according to the manufacturer’s instruction. Briefly, purified CD4^+^ T cells were incubated with CFSE at 37°C for 5 min. After quenching the staining by the addition of prewarmed culture media, the cells were incubated for 5 min at room temperature, followed by the collection and washing of cells. The cells were cocultured under Th17‐polarizing conditions and Treg‐polarizing conditions for 4 days. Graphical displays showing each cell division were obtained using FlowJo software.

### 2.9. Histological Analysis and Immunohistochemistry Staining

Paraffin‐embedded skin sections (4 μm) underwent hematoxylin and eosin (H&E) staining for acanthosis quantification or immunohistochemical staining with Ki‐67 (Cell Signaling, D3B5, 1:200) and phosphorylated STAT3 (p‐STAT3) (Cell Signaling, D3A7, 1:100). All the sections were counterstained with hematoxylin. Images were captured using a microscope (Hamamatsu, Japan). Ki‐67 and p‐STAT3 nuclear staining was quantified based on the number of positive cells per square millimeter of the intraepidermal area. Positive cell density was calculated from five 400× fields per sample using ImageJ v1.53 (NIH).

### 2.10. Intracellular Uptake of hucMSC‐Exos

hucMSC‐Exos were labeled with red fluorescent dye (DiD) (Beyotime, China). For the internalization assay, DiD‐labeled hucMSC‐Exos were cocultured with RAW264.7 cells for 12 h. The cells were then washed with PBS and fixed with 4% paraformaldehyde. Nuclei were stained with DAPI (Beyotime, China), and F‐actin was stained with Actin‐Tracker Green (Beyotime, China) according to the manufacturer’s instructions. The internalization of hucMSC‐Exos was observed using a laser scanning confocal microscope.

### 2.11. Dual‐Luciferase Reporter Assay

The 3′‐UTR sequence of the mutated STAT3 and the predicted target site were inserted into the pmir‐GLO‐promoter vector (Promega, USA). They were named pmirGLO‐STAT3‐WT and pmirGLO‐STAT3‐MUT, respectively. HEK293T cells transfected with miR‐NC or miR‐125b‐5p mimic were seeded into 96‐well plates and co‐transfected with 100 ng of pmirGLO‐STAT3‐WT or pmirGLO‐STAT3‐MUT. In the next experiment, WT or mutated STAT3‐binding site reporter vectors were obtained from Obio Technology (TSingke, China) and transfected into HEK293T cells. The luciferase activity in the transfected cells was measured using a dual luciferase reporter assay system (Vazyme, China). The relative expression of firefly luciferase activity was normalized to Renilla luciferase activity.

### 2.12. Statistical Analysis

All statistical analyses were performed using GraphPad Prism Version 8.0.1 (GraphPad Software, USA). Data are presented as the mean ± standard error of the mean (SEM) unless otherwise indicated. Comparisons between two independent groups were performed using two‐sided Student’s *t*‐tests, whereas comparisons among three or more independent groups were performed using one‐way analysis of variance (ANOVA) followed by Tukey’s multiple‐comparisons test, as appropriate. Longitudinal PASI‐like scores were analyzed using two‐way repeated‐measures ANOVA with the treatment group and time as factors. Sphericity was not assumed, and the Geisser–Greenhouse correction was applied. Pairwise comparisons among treatment groups within individual time points were performed using Tukey’s multiple‐comparisons test. All statistical tests were two‐sided, and the a priori significance level was set at *α* = 0.05. Comparisons among predefined experimental groups and their corresponding phenotypic and molecular endpoints were considered planned analyses, whereas miRNA‐sequencing analysis was exploratory and was used to identify candidate miRNAs for subsequent targeted validation. No subgroup analyses were performed. Asterisks in the figures indicate statistically significant pairwise comparisons, as specified in the corresponding figure legends.

## 3. Results

### 3.1. The Characterization of hucMSCs

**Figure 1:**
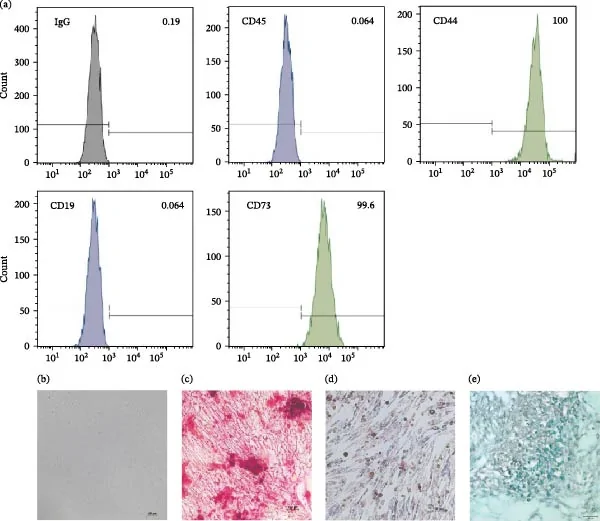
Phenotypic characterization of human umbilical cord mesenchymal stem cells (hucMSCs). (a) Flow cytometric immunophenotyping demonstrating canonical MSC surface marker expression profiles. Histograms depict high positivity for CD73 and CD44 (green), with isotype controls (gray). Hematopoietic lineage exclusion was confirmed by minimal detection of CD19 and CD45(blue). (b) Phase‐contrast micrograph illustrating typical spindle‐shaped morphology of passage 3 hucMSCs (scale bar: 200 μm). (c–e) Functional validation of trilineage differentiation capacity. (c) Mineralized matrix deposition after15‐day osteogenic induction (alizarin red S staining; scale bar: 100 μm). (d) Lipid droplet accumulation following 14‐day adipogenic induction (oil red O staining; scale bar: 100 μm). (e) Sulfated glycosaminoglycan synthesis post 20‐day chondrogenic induction (alcian blue staining; scale bar: 50 μm).

hucMSCs were characterized through comprehensive immunophenotypic profiling and trilineage differentiation assays. Flow cytometric analysis displayed a mesenchymal‐like immunophenotype, demonstrating high expression of MSC markers CD73 (99.5% ± 0.3%) and CD44 (100% ± 0.1%) while exhibiting negligible hematopoietic lineage contamination (CD19: 0.45% ± 0.1%; CD45: 0.79% ± 0.2%) (Figure 1a). Primary cultures displayed a characteristic spindle‐shaped fibroblastoid morphology under phase contrast microscopy (Figure 1b). Multipotency was validated through directed differentiation protocols: osteogenic induction precipitated calcium deposition detectable by alizarin red S staining (Figure 1c), adipogenic commitment resulted in intracellular lipid vacuole formation visualized with oil red O (Figure 1d), and chondrogenic differentiation produced proteoglycan‐rich extracellular matrix confirmed by alcian blue histochemistry (Figure 1e).

### 3.2. Exosome Purification, Characterization, and Internalization Into Cells

**Figure 2:**
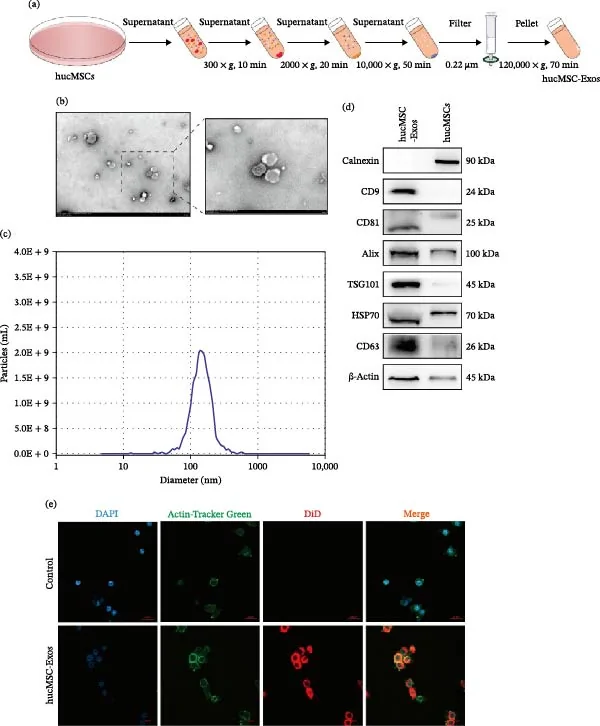
Isolation and characterization of hucMSC‐derived exosomes (hucMSC‐Exos). (a) Schematic workflow of exosome isolation through sequential centrifugation protocol. (b) Representative TEM micrograph demonstrating characteristic exosomal morphology with intact lipid bilayers (scale bar: 200 nm). (c) Particle size distribution of the hucMSC‐Exos measured by nanoparticle tracking analysis (NTA). (d) Western blot analysis of exosome‐positive markers (CD9, CD63, CD81, HSP70, and TSG101) and exosome negative marker (calnexin) of hucMSC‐Exos compared with hucMSCs lysis. (e) Confocal micrographs of RAW264.7 macrophages internalizing DiD‐labeled exosomes (red) after 12 hr coculture. Nuclei counterstained with DAPI (blue). F‐actin cytoskeleton with Actin‐Tracker Green (green) (scale bar: 20 μm).

hucMSC‐Exos were isolated via differential ultracentrifugation (10,000 ×*g* debris removal followed by 120,000 ×*g* ultracentrifugation) from conditioned media of passages 3–6 cultures (Figure 2a). Structural characterization using TEM revealed classical exosomal ultrastructure with intact bilayer membranes exhibiting a characteristic cup‐shaped morphology (Figure 2b). Quantitative NTA demonstrated monodisperse populations with a median hydrodynamic diameter of 142.2 ± 3.5 nm at a concentration of 2.1 × 10^9^ particles/mL (Figure 2c). Immunoblotting confirmed enrichment of exosomal tetraspanins (CD9, CD63, and CD81), ESCRT components (TSG101, Alix), and stress protein HSP70 while excluding endoplasmic reticulum contaminant calnexin (Figure 2d). Cellular internalization dynamics was investigated using dual‐fluorescence labeling. DiD‐labeled hucMSC‐Exos (1 μM, 37°C, 15 min) were cocultured with RAW264.7 macrophages for 12 h prior to cytoskeletal fixation (4% paraformaldehyde, 30 min) and permeabilization (0.1% Triton X‐100, 30 min). Cells were subsequently stained with Actin‐Tracker Green for F‐actin visualization and DAPI for nuclear counterstaining (37°C, 30 min). Confocal microscopy demonstrated exosomal accumulation in the perinuclear region with partial actin filament association (Figure 2e).

### 3.3. hucMSC‐Exos Ameliorate IMQ‐Induced Psoriasiform Dermatitis in Mice

**Figure 3:**
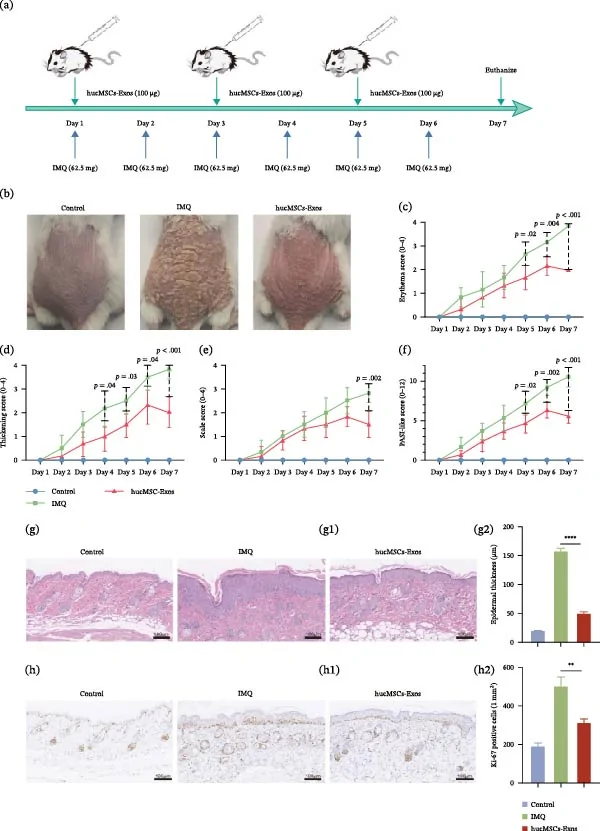
Therapeutic effects of hucMSC‐Exos on IMQ‐induced psoriasiform dermatitis. (a) Experimental timeline: male BALB/c mice (6–8 weeks) received daily topical IMQ (5% cream, 62.5 mg) on shaved dorsum for six consecutive days. hucMSC‐Exos (100 μg) or vehicle (PBS) were administered subcutaneously on days 1, 3, and 5. (b) Representative macroscopic images showing dorsal skin phenotypes at day 7. (c–f) Longitudinal PASI‐like scoring: (c) erythema score, (d) thickening score, (e) scaling score, and (f) composite PASI‐like score. Data are presented as mean ± SEM (*n* = 6 mice per group). Longitudinal scores were analyzed using two‐way repeated‐measures ANOVA with treatment group and time as factors. Sphericity was not assumed, and the Geisser–Greenhouse correction was applied. Pairwise comparisons among treatment groups within each time point were performed using Tukey’s multiple‐comparisons test. Adjusted *p* values are shown for the indicated comparisons between the IMQ and hucMSC‐Exos groups. (g) Histomorphometric analysis: (g1) H&E‐stained paraffin sections (scale bar: 100 μm); (g2) quantification of epidermal thickness (five random fields/sample). (h) Keratinocyte proliferation assessment: (h1) representative immunohistochemical images of Ki‐67‐positive cells (brown, DAB chromogen; scale bar: 100 μm); (h2) quantitative analysis of Ki67 labeling index (five random fields/sample). For the endpoint analyses in subfigures (g) and (h), comparisons among groups were performed using one‐way ANOVA followed by Tukey’s multiple‐comparisons test;  ^∗^ *p* < 0.05,  ^∗∗^ *p* < 0.01,  ^∗∗∗^ *p* < 0.001,  ^∗∗∗∗^ asterisks indicate statistical significance vs. IMQ group.

The therapeutic effects of hucMSC‐Exos were evaluated in a widely used IMQ‐induced murine model of psoriasiform dermatitis (*n* = 6/group). Subcutaneous administration of hucMSC‐Exos (100 μg in 200 μL PBS per dose) was performed on days 1, 3, and 5 post‐IMQ induction (Figure 3a). As expected, the PBS‐control mice developed typical psoriasis‐like lesions with evident clinical and pathological changes. Macroscopic evaluation revealed marked attenuation of psoriasiform features in exosome‐treated cohorts, including reduced erythema, scaling, and epidermal hyperplasia, compared to IMQ controls (Figure 3b). Longitudinal PASI‐like scoring revealed significant treatment‐by‐time interactions for erythema, thickening, scaling, and composite PASI‐like scores (erythema: *F* (12, 90) = 37.86; thickening: *F* (12, 90) = 34.19; scaling: *F* (12, 90) = 21.58; composite score: *F* (12, 90) = 72.98; all *p* < 0.001; Figure 3c–f). Post hoc comparisons showed that hucMSC‐Exos‐treated mice had lower erythema scores than IMQ‐treated mice from Day 5 onward, lower thickening scores from Day 4 onward, and lower scaling scores on Day 7. The composite PASI‐like score was significantly lower in the hucMSC‐Exos group from Day 5 onward. On Day 7, the mean differences between the IMQ and hucMSC‐Exos groups were 1.83 points for erythema (95% CI, 1.29–2.38; adjusted *p* < 0.001), 1.83 points for thickening (95% CI, 0.97–2.70; adjusted *p* < 0.001), 1.33 points for scaling (95% CI, 0.56–2.11; adjusted *p* = 0.002), and 5.00 points for the composite PASI‐like score (95% CI, 3.30–6.70; adjusted *p* < 0.001).(Figure 3c–f).

Histopathological analysis of H&E‐stained sections (4 μm thickness) confirmed therapeutic effects, showing a 68.07% reduction in acanthosis (Figure 3g). Immunohistochemical profiling revealed 37.75% suppression of Ki‐67+ proliferating keratinocytes, indicating the normalization of epidermal turnover (Figure 3h). These findings demonstrate that hucMSC‐Exos ameliorate epidermal hyperplasia and inflammatory features in the IMQ‐induced psoriasiform dermatitis model.

### 3.4. hucMSC‐Exos Attenuate Inflammation in the IMQ‐Induced Psoriasiform Dermatitis Model

**Figure 4:**
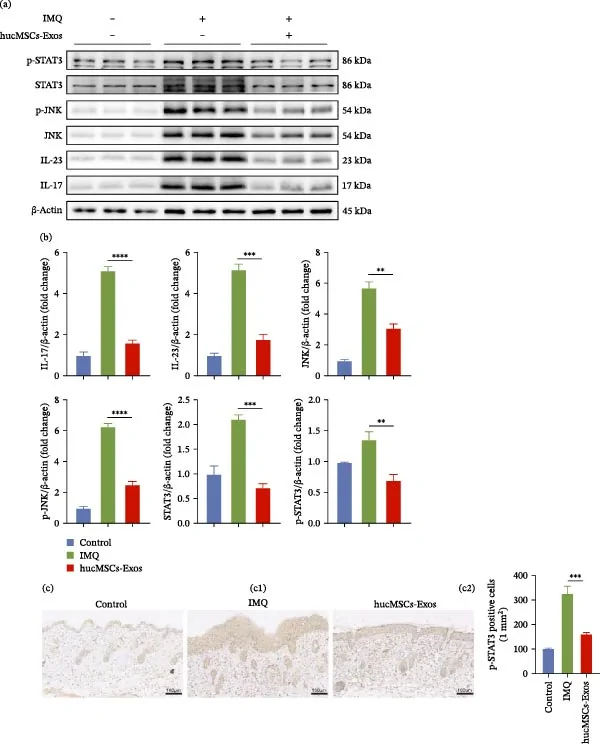
hucMSC‐Exos reduce phosphorylated JNK and phosphorylated STAT3 levels in the IMQ‐induced psoriasiform dermatitis model. (a) Western blot analysis of key signaling proteins in dorsal skin homogenates. Antibodies: p‐JNK, JNK, p‐STAT3, STAT3, IL‐23, IL‐17 A, β‐actin. (b) Densitometric quantification normalized to β‐actin (ImageJ v1.53). Data expressed as mean fold‐change ± SEM vs. control. Statistical analyses are performed by one‐way ANOVA with Tukey’s post hoc test;  ^∗^ *p* < 0.05,  ^∗∗^ *p* < 0.01,  ^∗∗∗^ *p* < 0.001,  ^∗∗∗∗^ *p* < 0.0001 vs. IMQ group. (c) (c1) Representative IHC images of p‐STAT3 (brown) counterstained with hematoxylin (scale bar: 100 μm). (c2) Quantitative analysis of p‐STAT3 labeling index. Data are presented as the mean ± SEM. Statistical analyses were performed using one‐way ANOVA followed by Tukey’s post hoc test.  ^∗^ *p* < 0.05,  ^∗∗^ *p* < 0.01,  ^∗∗∗^ *p* < 0.001,  ^∗∗∗∗^ asterisks indicate statistical significance vs. IMQ group.

We next examined the effects of hucMSC‐Exos on JNK and STAT3 phosphorylation in lesional skin. Western blot analysis demonstrated significantly reduced levels of phosphorylated JNK (p‐JNK) and p‐STAT3 in hucMSC‐Exos‐treated mice compared with the IMQ group, whereas total JNK and STAT3 levels remained unchanged (Figure 4a,b). Immunohistochemical quantification confirmed a 50.39% reduction in nuclear p‐STAT3‐positive keratinocytes (Figure 4c). Concomitantly, IL‐23 and IL‐17A protein levels were decreased relative to those in the IMQ group, suggesting attenuation of the IL‐23/Th17 inflammatory axis (Figure 4a,b).

**Figure 5:**
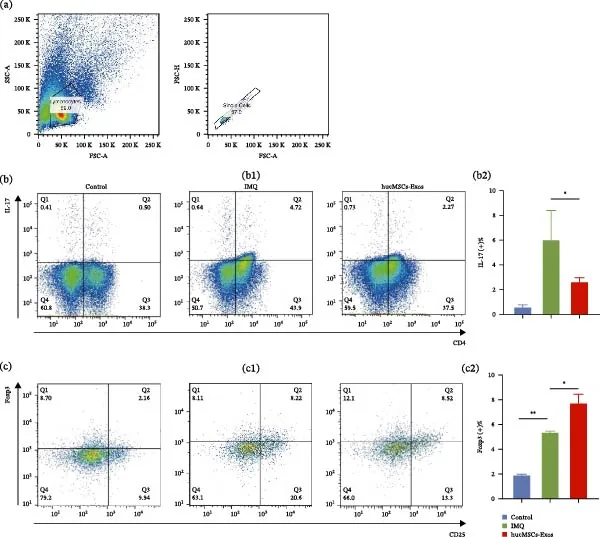
hucMSC‐Exos modulate Th17 and Treg cell frequencies in vivo. (a–c) Splenic Th17 and Treg cell frequencies. (a) Flow cytometry gating strategy for analysis. (b) (b1) Representative figures of Th17 cells from a healthy control, IMQ, and IMQ + hucMSC‐Exos group. CD4^+^ IL‐17^+^ Th17 were gated from the CD4^+^ subset T cells. (b2) Quantification of CD4^+^ IL‐17A^+^ Th17‐cell frequencies (*n* = 3/group). (c) (c1) Representative figures of Treg cells from a healthy control, IMQ, and IMQ + hucMSC‐Exos group. CD4^+^CD25^+^Foxp3^+^ Treg were gated from the CD4+ subset T cells. (c2) Quantification of CD4^+^CD25^+^Foxp3^+^ Treg‐cell frequencies (*n* = 3/group). Data are presented as the mean ± SEM. Statistical analyses were performed using one‐way ANOVA followed by Tukey’s post hoc test.  ^∗^ *p* < 0.05,  ^∗∗^ *p* < 0.01,  ^∗∗∗^ *p* < 0.001,  ^∗∗∗∗^ asterisks indicate statistical significance.

Then, splenic CD4^+^ T cells were obtained to assess the effects of hucMSC‐Exos on Th17/Treg imbalance in the IMQ‐induced psoriasiform dermatitis model. Flow cytometric analysis of splenocytes showed that hucMSC‐Exos treatment decreased the frequency of CD4^+^IL‐17 A^+^ Th17 cells compared with the IMQ group (Figure 5a,b). In parallel, the frequency of CD4^+^CD25^+^Foxp3^+^ Treg cells was increased following hucMSC‐Exos treatment (Figure 5c). These findings indicate that hucMSC‐Exos modulate Th17 and Treg cell populations in the IMQ‐induced psoriasiform dermatitis model.

**Figure 6:**
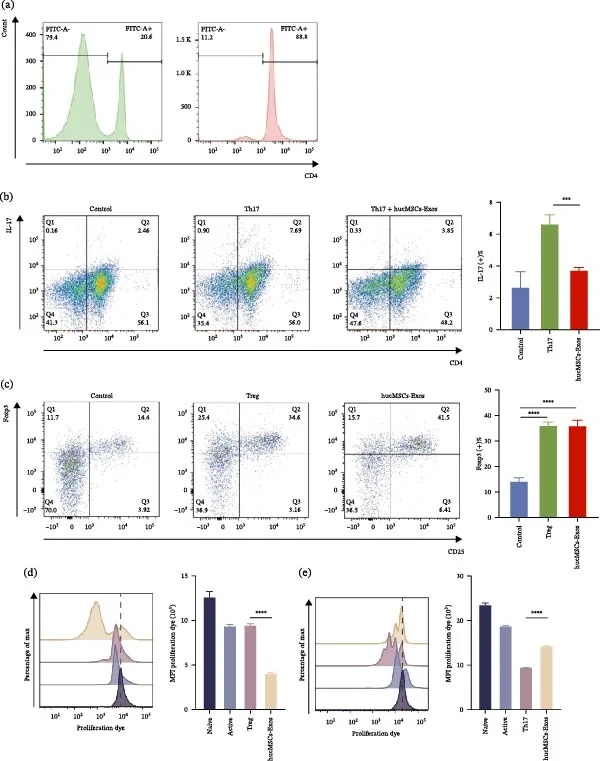
Effects of hucMSC‐Exos on Th17 and Treg polarization and proliferation in vitro. (a) CD4^+^ T cell isolation: flow verification of CD4^+^ population purity. (b) CD4^+^ T cells were activated under Th17 conditions in the presence or absence of hucMSC‐Exos for 72 h. Representative plots are gated on CD4^+^ IL‐17A^+^ T cells and graphs show the expression of IL‐17A (*n* = 3/group). (c) CD4^+^ T cells were activated under Treg conditions or in the presence of hucMSC‐Exos for 72 h. Representative plots are gated on CD4^+^CD25^+^Foxp3^+^ T cells and graphs show the expression of Foxp3 (*n* = 3/group). (d) Proliferation of CD4+ T cells cultured under Treg conditions or in the presence of hucMSC‐Exos. Representative histogram overlay shows the proliferation of live CD4 T cells on day 4 (*n* = 3/group). (e) Proliferation of CD4^+^ T cells cultured under Th17 conditions in the presence or absence of hucMSC‐Exos. Representative histogram overlay shows the proliferation of live CD4^+^ T cells on day 4 (*n* = 3/group). For subfigures (b) and (e), comparisons were made with the Th17 group. For subfigures (c) and (d), comparisons were made with the Treg group. Data are presented as the mean ± SEM. Statistical analyses were performed using one‐way ANOVA followed by Tukey’s post hoc test.  ^∗^ *p* < 0.05,  ^∗∗^ *p* < 0.01,  ^∗∗∗^ *p* < 0.001,  ^∗∗∗∗^ asterisks indicate statistical significance.

In vitro isolation using the MojoSort Mouse CD4^+^ T Cell Isolation Kit demonstrated a purity of 88.8% in the CD4^+^ T cell population (Figure 6a). Furthermore, hucMSC‐Exos suppressed Th17 polarization and proliferation, as evidenced by a decreased proportion of IL‐17 A^+^ cells and increased fluorescence intensity of proliferation dye (Figure 6b,e). However, the addition of hucMSC‐Exos to CD4^+^ T cells significantly enhanced Treg polarization and proliferation, as evidenced by an increased proportion of Foxp3^+^ cells and reduced fluorescence intensity of the proliferation dye (Figure 6c,d). Together, these findings show that hucMSC‐Exos treatment is associated with reduced phosphorylation of JNK and STAT3 and changes in Th17 and Treg cell responses.

### 3.5. Identification of miR‐125b‐5p in hucMSC‐Exos and Validation of STAT3 as a Direct Target

**Figure 7:**
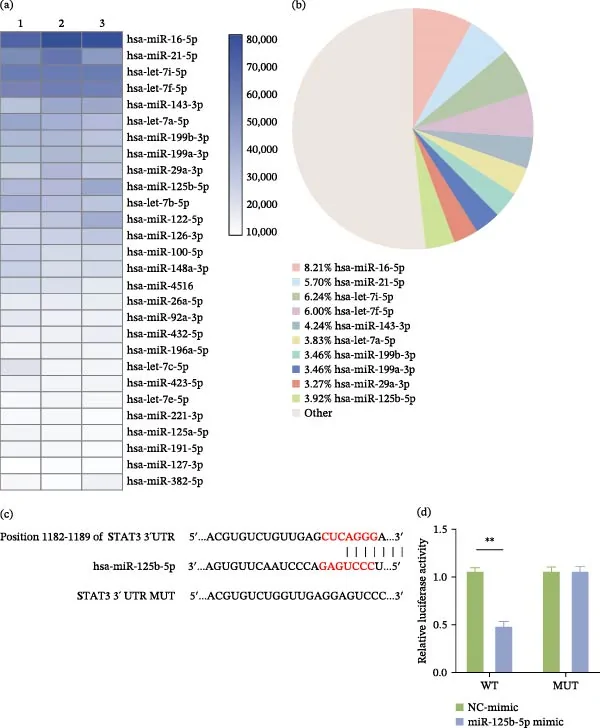
Identification of miR‐125b‐5p in hucMSC‐Exos and validation of STAT3 as a direct target. (a) Heat map of the most abundant miRNAs across three biological replicates in hucMSC‐Exos by miRNA‐seq. (b) Relative percentage of top ten miRNAs in total miRNA reads. (c) Schematic illustration of the predicted miR‐125b‐5p‐binding site within the 3′‐UTR of STAT3. The underlined sequences indicate the mutated seed matching region. (d) Dual‐luciferase reporter assay validating STAT3 as a direct target of miR‐125b‐5p (*n* = 3/group). WT, wild type; MUT, mutant. Data are presented as the mean ± SEM. Statistical analyses were performed using one‐way ANOVA followed by Tukey’s post hoc test.  ^∗^ *p* < 0.05,  ^∗∗^ *p* < 0.01,  ^∗∗∗^ *p* < 0.001,  ^∗∗∗∗^ asterisks indicate statistical significance.

miRNA sequencing revealed a diverse miRNA cargo in hucMSC‐Exos. The 10 most abundant miRNAs collectively accounted for ~49% of the total miRNA reads and included miR‐16‐5p, miR‐21‐5p, miR‐125b‐5p, miR‐143‐3p, and miR‐199b‐3p (Figure 7a,b). Based on its abundance in our sequencing dataset and its previously reported involvement in psoriasis‐associated keratinocyte biology, miR‐125b‐5p was selected for further mechanistic investigation. Bioinformatic analysis using TargetScan v7.2 and miRanda‐mirSVR identified a conserved putative miR‐125b‐5p‐binding site within the STAT3 3′ UTR (positions 1182–1189) (Figure 7c). Dual‐luciferase reporter assays in HEK293T cells showed that miR‐125b‐5p reduced the luciferase activity of the STAT3 3′ UTR wild‐type reporter, whereas mutation of the predicted binding site abolished this effect (Figure 7d). These results support STAT3 as a direct target of miR‐125b‐5p in our experimental system.

### 3.6. Functional Inhibition of Exosomal miR‐125b‐5p Attenuates the Therapeutic Effects of hucMSC‐Exos in IMQ‐Induced Psoriasiform Dermatitis

**Figure 8:**
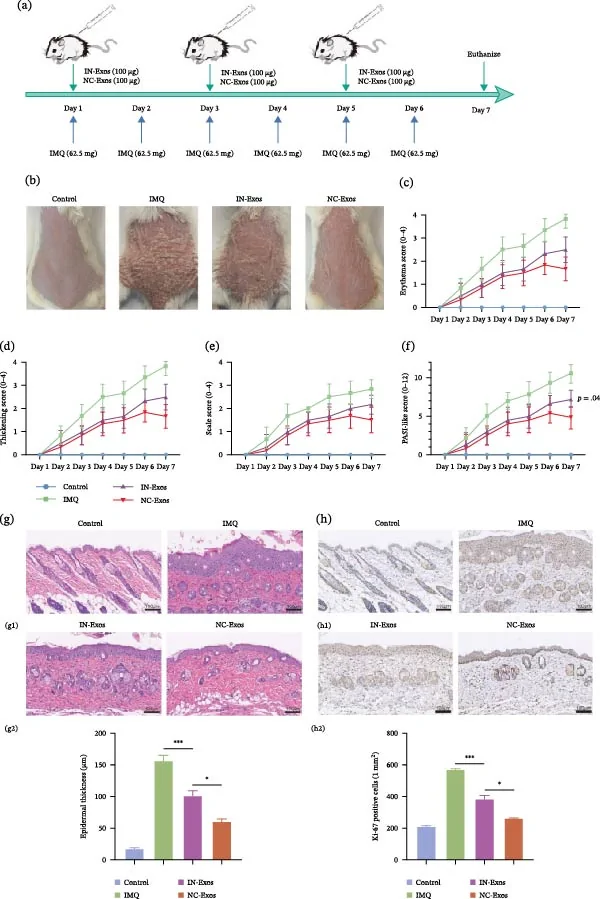
Functional inhibition of exosomal miR‐125b‐5p reduces the therapeutic effects of hucMSC‐Exos. (a) Experimental timeline: male BALB/c mice (6–8 weeks) received daily topical IMQ (5% cream, 62.5 mg) on shaved dorsum for six consecutive days. IN‐Exos (100 μg Exos + 20 pmoL miR‐125b‐5p inhibitor), NC‐Exos (100 μg Exos + 20 pmoL negative control) were administered subcutaneously on days 1, 3, and 5. (b) Representative macroscopic images showing dorsal skin phenotypes at day 7. (c–f) Longitudinal PASI‐like scoring: (c) erythema score, (d) thickening score, (e) scaling score, and (f) composite PASI‐like score. Data are presented as mean ± SEM (*n* = 6 mice per group). Longitudinal scores were analyzed using two‐way repeated‐measures ANOVA with treatment group and time as factors. Sphericity was not assumed, and the Geisser–Greenhouse correction was applied. Pairwise comparisons among treatment groups within each time point were performed using Tukey’s multiple‐comparisons test. Adjusted *p* values are shown for the indicated comparisons. (g) Histomorphometric analysis: (g1) H&E‐stained paraffin sections (scale bar: 100 μm); (g2) quantification of epidermal thickness (five random fields/sample). (h) Keratinocyte proliferation assessment: (h1) representative immunohistochemical images of Ki‐67‐positive cells (brown, DAB chromogen; scale bar: 100 μm); (h2) quantitative analysis of Ki‐67 positive cells (five random fields/sample). For the endpoint analyses in panels (g) and (h), comparisons among groups were performed using one‐way ANOVA followed by Tukey’s multiple‐comparisons test;  ^∗^ *p* < 0.05,  ^∗∗^ *p* < 0.01,  ^∗∗∗^ *p* < 0.001,  ^∗∗∗∗^ asterisks indicate statistical significance vs. IMQ group.

To evaluate whether exosomal miR‐125b‐5p contributes to the therapeutic activity of hucMSC‐Exos, we compared miR‐125b‐5p IN‐Exos with negative control‐loaded exosomes in the IMQ‐induced psoriasiform dermatitis model. The IMQ‐treated mice were injected with PBS, IN‐Exos (100 μg in 200 μL PBS per dose), or NC‐Exos (100 μg in 200 μL PBS per dose) subcutaneously on days 1, 3, and 5 post‐IMQ induction. The mice were euthanized on day 7 (Figure 8a). Compared with NC‐Exos, IN‐Exos exhibited a reduced capacity to alleviate IMQ‐induced psoriasiform dermatitis (Figure 8b). Longitudinal PASI‐like scoring showed generally higher erythema, thickening, scaling, and composite scores in the IN‐Exos group than in the NC‐Exos group (Figure 8c–f). However, direct Tukey‐adjusted post hoc comparisons between IN‐Exos and NC‐Exos did not reach statistical significance for erythema, thickening, or scaling scores at the individual time points examined. For the composite PASI‐like score, no significant difference was detected between the two groups on Days 2–6, whereas on Day 7 the score was higher in the IN‐Exos group than in the NC‐Exos group (mean difference, 2.33 points; 95% CI, 0.06–4.60; adjusted *p* = 0.04; Figure 8f). These findings indicate that inhibition of exosomal miR‐125b‐5p was associated with a modest reduction in the clinical efficacy of hucMSC‐Exos, which became statistically evident in the composite PASI‐like score at the final assessment.

Histological analysis further showed that functional inhibition of exosomal miR‐125b‐5p attenuated the ability of NC‐Exos to reduce epidermal thickness and Ki‐67‐positive keratinocyte abundance (Figure 8g,h).

**Figure 9:**
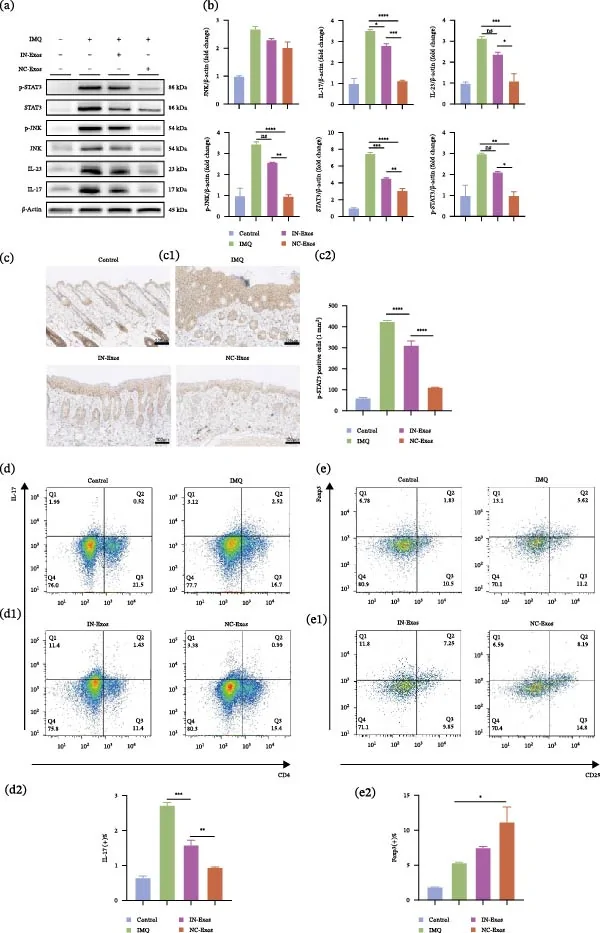
Functional inhibition of exosomal miR‐125b‐5p is associated with increased STAT3 and JNK phosphorylation and altered Th17 and Treg cell frequencies. (a) Western blot analysis of key signaling proteins in dorsal skin homogenates. Lanes: 1‐control, 2‐IMQ, 3‐IMQ + IN‐Exos, 4‐IMQ + NC‐Exos. Antibodies: p‐JNK, JNK, p‐STAT3, STAT3, IL‐23, IL‐17 A, β‐actin. (b) Densitometric quantification normalized to β‐actin (ImageJ v1.53) (*n* = 3/group). Data expressed as mean fold‐change ± SEM vs. control. Statistical analyses were performed using one‐way ANOVA followed by Tukey’s post hoc test.  ^∗^ *p* < 0.05,  ^∗∗^ *p* < 0.01,  ^∗∗∗^ *p* < 0.001,  ^∗∗∗∗^ asterisks indicate statistical significance. (c) (c1) Representative IHC images of p‐STAT3 (brown) counterstained with hematoxylin (scale bar: 100 μm). (c2) Quantitative analysis of p‐STAT3 labeling index (five random fields/sample). (d, e) Splenic Th17 and Treg cell frequencies. (d) (d1) Representative figures of Th17 cells from a control, IMQ, IMQ + IN‐Exos, and IMQ + NC‐Exos group. CD4^+^ IL‐17^+^ Th17 were gated from the CD4+ subset T cells. (d2) Quantification of CD4^+^ IL‐17A^+^ Th17‐cell frequencies (*n* = 3/group). (e) (e1) Representative figures of Treg cells from a control, IMQ, IMQ + IN‐Exos, and IMQ + NC‐Exos group. CD4^+^CD25^+^Foxp3^+^ Treg were gated from the CD4+ subset T cells. (e2) Quantification of CD4^+^CD25^+^Foxp3^+^ Treg‐cell frequencies (*n* = 3/group). Data are presented as the mean ± SEM. Statistical analyses were performed using one‐way ANOVA followed by Tukey’s post hoc test.  ^∗^ *p* < 0.05,  ^∗∗^ *p* < 0.01,  ^∗∗∗^ *p* < 0.001,  ^∗∗∗∗^ asterisks indicate statistical significance.

Compared with NC‐Exos, IN‐Exos treatment was associated with increased phosphorylation of STAT3 and JNK, together with higher IL‐23 and IL‐17A expression in the lesional skin (Figure 9a,b). Quantitative analysis of p‐STAT3‐positive cells further showed increased nuclear p‐STAT3 staining in the IN‐Exos group relative to the NC‐Exos group (Figure 9c). Compared with NC‐Exos, IN‐Exos treatment increased the frequency of CD4^+^IL‐17 A^+^ Th17 cells and decreased the frequency of CD4^+^CD25^+^Foxp3^+^ Treg cells in the spleen (Figure 9d,e). Together, these findings suggest that exosomal miR‐125b‐5p contributes to the therapeutic activity of hucMSC‐Exos, at least in part through the regulation of STAT3‐associated inflammatory signaling.

## 4. Discussion

Psoriasis is one of the most common inflammatory diseases, characterized by scaly erythematous plaques resulting from keratinocyte hyperproliferation and immune dysregulation [2]. As a chronic T cell‐mediated autoimmune disorder, its pathogenesis hinges on the imbalance between pro‐inflammatory Th1/Th17 and anti‐inflammatory Th2/Treg responses [18]. Central to this process is the IL‐23/Th17 axis, where IL‐23 activates STAT3 signaling to promote Th17 differentiation and sustain their survival [33–36]. These Th17 cells secrete IL‐17A/F, which directly drive inflammatory cascades through neutrophil recruitment, antimicrobial peptide overexpression, and epidermal hyperplasia—pathological hallmarks underlying the clinically observed plaques [37, 38]. Despite advances in biologic therapies targeting IL‐17 or IL‐23, clinical efficacy remains suboptimal due to high relapse rates post‐treatment cessation and interpatient variability in drug response [39–41].

MSCs, known for their immunosuppressive and anti‐inflammatory properties [42], hold therapeutic promise due to their low immunogenicity and differentiation potential [43]. In addition, several clinical studies have demonstrated the promising therapeutic effects of various types of MSCs in psoriasis treatment [44–46]. However, MSC therapy faces challenges, such as the risk of unintended differentiation, immune reactions, and viral transmission in allogeneic transplants [47, 48]. More recently, MSC‐derived exosomes have attracted considerable attention as cell‐free therapeutic products because they retain key paracrine activities while avoiding several practical limitations of viable cell therapies [49, 50]. Unlike live MSC products, exosome‐based preparations do not require preservation of cell viability during administration and can potentially facilitate standardized dosing, off‐the‐shelf storage, transport, and repeated outpatient administration. Their acellular nature may also reduce risks associated with viable cell delivery, such as microvascular obstruction, ectopic engraftment, and unpredictable cellular persistence. Nevertheless, exosome isolation, purification, and quality control remain technically demanding and may add to manufacturing costs. Thus, their translational advantages lie primarily in standardization, storage and logistical flexibility, dosing convenience, and avoidance of viable‐cell‐associated risks rather than in universally simpler manufacturing.

Previous studies have shown that MSC‐derived exosomes can modulate immune cell responses, including the functions of macrophages and T cells [51]. Related MSC‐based cell‐free approaches, such as conditioned media, have also demonstrated anti‐inflammatory potential, although these preparations differ from purified exosomes in their composition and mechanisms of action [52].

In this study, hucMSC‐Exos administration significantly attenuated psoriasis‐like clinical and histopathological features in the IMQ‐induced murine model, including reduced PASI‐like scores, epidermal acanthosis, and inflammatory changes. These findings are consistent with previous studies showing that hucMSC‐Exos alleviate psoriasis‐like inflammation through modulation of the IL‐23/IL‐17 axis, inhibition of STAT3 activation, and regulation of dendritic cell function [17]. Building on these observations, our study identifies exosomal miR‐125b‐5p as one contributor to the therapeutic activity of hucMSC‐Exos rather than as an exclusive mediator of their effects. These results are similar to those of previous studies, but the underlying mechanisms remain unclear [17, 53, 54]. The hallmark of psoriasis pathogenesis is the immune imbalance driven by Th17‐mediated chronic inflammation, exacerbated by defects in Treg function [55–57]. Notably, we observe elevated IL‐23 levels in psoriatic lesions and enhanced Th17 cell differentiation in the spleen, which is consistent with prior studies [58]. Our findings showed that hucMSC‐Exos reduced Th17 polarization and proliferation while promoting Treg polarization and proliferation in vitro. Consistently, hucMSC‐Exos treatment was associated with lower Th17‐cell frequencies and higher Treg‐cell frequencies in the IMQ‐induced psoriasiform dermatitis model. These observations suggest that modulation of Th17 and Treg cell responses may contribute to the immunomodulatory effects of hucMSC‐Exos. However, MSC‐derived exosomes contain diverse bioactive cargos, and their immunological effects may vary depending on the MSC source, exosome composition, experimental conditions, and disease context. At the molecular level, the Th17/Treg imbalance is intricately regulated by inflammatory signaling pathways. A prior study [59] showed that JNK activation promotes the secretion of inflammatory cytokines such as TNF‐α, IL‐1β, IL‐6, and IL‐17, which drive leukocyte infiltration and tissue damage. Additionally, the STAT3 pathway plays a central role in modulating the IL‐23/IL‐17 axis, facilitating Th17 cell differentiation and keratinocyte proliferation [31]. Previous studies have demonstrated that STAT3 can integrate signals from multiple upstream kinases. For example, in systemic sclerosis fibroblasts, JNK, together with other kinases such as JAK and SRC, has been implicated in STAT3 activation [60]. However, this relationship is context‐dependent and has not been established as a universal linear JNK–STAT3 pathway in psoriasis. In the present study, increased phosphorylation of both JNK and STAT3 was observed in the lesional skin, whereas hucMSC‐Exos treatment reduced the activation of both pathways. These concurrent changes suggest that hucMSC‐Exos modulate multiple inflammatory signaling pathways; however, our data do not establish a direct causal relationship between JNK and STAT3.

More broadly, nanoscale therapeutic systems have been shown to modulate inflammatory signaling through diverse molecular pathways. For example, silymarin/silybin‐functionalized selenium nanoparticles attenuated LPS‐induced inflammatory responses through inhibition of PI3K/AKT/NF‐κB signaling, illustrating that nanoparticle‐based interventions can regulate interconnected inflammatory networks [61]. In a psoriasis‐like model, exosome‐mediated delivery of epigallocatechin‐3‐gallate was also reported to modulate inflammatory cytokines, NF‐κB, and apoptosis‐related signaling, supporting the potential of exosome‐based delivery systems to influence multiple inflammatory processes [62]. These studies provide complementary evidence for the broader anti‐inflammatory potential of nanoscale and exosome‐based therapeutic strategies; however, the specific JNK/STAT3‐ and Th17/Treg‐associated changes observed in our study are supported primarily by our experimental data and pathway‐specific literature.

miRNAs carried by EVs can regulate multiple target genes in a context‐dependent manner. In psoriasis and other cutaneous settings, miR‐125 b has been reported to regulate targets such as FGFR2, indicating that its biological effects are unlikely to be mediated by a single target. In the present study, miRNA sequencing identified miR‐125b‐5p as one of several abundant miRNAs in hucMSC‐Exos. Based on bioinformatic prediction and subsequent dual‐luciferase reporter validation, we identified STAT3 as a direct target of miR‐125b‐5p in our experimental system. Thus, our findings extend rather than exclude previously reported miR‐125 b target networks and do not imply that STAT3 is the sole target of miR‐125b‐5p or that miR‐125b‐5p is the dominant functional cargo of hucMSC‐Exos. Previous studies have reported reduced miR‐125 b expression in psoriatic skin and demonstrated its involvement in the regulation of keratinocyte proliferation and differentiation, including through targeting FGFR2 [22, 23]. Together with its relatively high abundance in our hucMSC‐Exo miRNA sequencing dataset, these observations prompted us to investigate miR‐125b‐5p as a candidate cargo molecule. The previously reported targeting of FGFR2 by miR‐125 b does not preclude additional context‐dependent targets. In the present study, bioinformatic prediction, followed by dual‐luciferase reporter analysis, supported STAT3 as a direct target of miR‐125b‐5p in our experimental system. Thus, our findings extend the reported target spectrum of miR‐125 b rather than suggesting that STAT3 is its exclusive target in psoriasis.

To further assess the functional relevance of exosomal miR‐125b‐5p, we prepared IN‐Exos and evaluated their effects in the IMQ‐induced psoriasiform dermatitis model. Compared with NC‐Exos, IN‐Exos showed a reduced capacity to improve cutaneous inflammation, epidermal hyperplasia, and Th17/Treg imbalance. This attenuation of therapeutic efficacy was accompanied by increased STAT3 phosphorylation and enhanced JNK activation. Collectively, these findings suggest that exosomal miR‐125b‐5p contributes, at least in part, to the anti‐inflammatory activity of hucMSC‐Exos through direct regulation of STAT3. The accompanying reduction in JNK activation should be interpreted as an associated signaling change because our current experiments do not establish JNK as a direct target of miR‐125b‐5p or define the causal hierarchy between JNK and STAT3. This mechanism does not exclude the involvement of other previously reported pathways, including TGF‐β2 signaling. Similar approaches have been used to investigate functional miRNA cargo in EVs in other disease settings [63].

**Figure 10:**
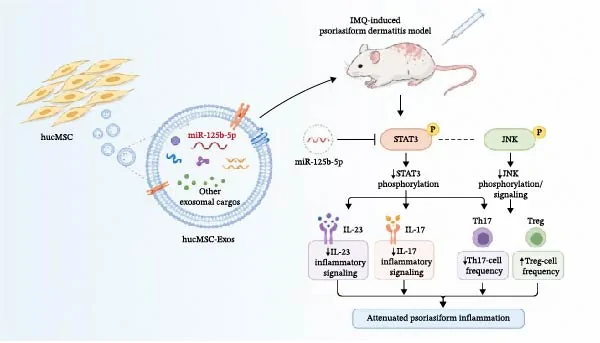
Proposed model of the contributory role of exosomal miR‐125b‐5p‐mediated STAT3 regulation in the effects of hucMSC‐Exos on IMQ‐induced psoriasiform dermatitis. The proposed model highlights direct regulation of STAT3 by exosomal miR‐125b‐5p. Changes in JNK phosphorylation and Th17/Treg‐associated immune responses are presented as associated effects rather than direct downstream consequences of miR‐125b‐5p. Other exosomal cargos and signaling pathways may also contribute to the overall therapeutic effects of hucMSC‐Exos.

Although previous studies have implicated TGF‐β2 signaling in the anti‐psoriatic effects of MSC‐derived exosomes [16], the TGF‐β2 pathway was not directly examined in the present study. Therefore, our findings should not be interpreted as excluding TGF‐β2‐dependent or other exosome‐mediated mechanisms. Instead, our data identify miR‐125b‐5p‐mediated regulation of STAT3 as one contributing mechanism underlying the therapeutic effects of hucMSC‐Exos. A schematic summary of the proposed contributory mechanism is shown in Figure 10.

Our study has several limitations. First, the functional interactions between miR‐125b‐5p and other exosomal miRNAs remain unclear and require further investigation. Second, the mechanistic relationship between STAT3 and JNK signaling needs to be clarified using pathway‐specific approaches. In particular, pathway‐specific inhibition or genetic perturbation will be required to determine whether JNK functions upstream of, parallel to, or independently from STAT3 in the context of hucMSC‐Exo treatment. Third, although the IMQ‐induced murine model reproduces several key inflammatory and histopathological features of psoriasis, it does not fully recapitulate the chronicity, complexity, and heterogeneity of human chronic plaque psoriasis. Therefore, the therapeutic and mechanistic findings obtained in this model should be interpreted within the limitations of an acute murine model and require validation in additional clinically relevant models. Finally, larger sample sizes and independent validation studies are needed to confirm the robustness of our findings. In addition, although previous studies have implicated TGF‐β2 signaling in the effects of MSC‐derived exosomes on psoriasis, this pathway was not directly evaluated in the present study. Future studies integrating TGF‐β2 and miRNA‐associated signaling will be necessary to define their relative and potentially complementary contributions.

In conclusion, our findings indicate that hucMSC‐Exos attenuate IMQ‐induced psoriasiform inflammation and modulate inflammatory signaling and Th17/Treg‐associated immune responses. Functional inhibition experiments further suggest that exosomal miR‐125b‐5p contributes, at least in part, to these effects through direct regulation of STAT3, with reduced JNK activation occurring in association with this regulatory process. This exosome‐based strategy offers distinct advantages over conventional biologics, including multi‐target modulation and reduced risk of systemic immunosuppression. Our findings position hucMSC‐Exos as a promising cell‐free therapeutic alternative for psoriasis and related Th17‐driven pathologies.

### 4.1. In Conclusion

The present study demonstrates that hucMSC‐Exos attenuate psoriasis‐like inflammation and are associated with modulation of inflammatory signaling and Th17/Treg‐associated immune responses in the IMQ‐induced psoriasiform dermatitis model. Exosomal miR‐125b‐5p was identified as one contributor to these effects and directly regulated STAT3 in our experimental system. Functional inhibition of miR‐125b‐5p reduced the therapeutic activity of hucMSC‐Exos and was accompanied by increased STAT3 phosphorylation, JNK activation, and changes in Th17 and Treg cell frequencies. These findings support a contributory role for miR‐125b‐5p‐mediated STAT3 regulation, while JNK modulation should be regarded as an associated signaling change whose mechanistic relationship with STAT3 requires further investigation.

## Author Contributions

Conceptualization: Qian Li, Ziyan Li, and Rui Ren. Data curation: Ziyan Li, Rui Ren, and Jieling Fu. Formal analysis, investigation, writing – original draft: Ziyan Li and Rui Ren. Methodology: Yunjia Wang, Jiaxin Huang, and Yuting Huang. Resources, supervision, writing – review and editing: Qian Li. Software, visualization: Jieling Fu and Kaiyuan Zhang. Validation: Zhichao Fan and Yijun Zhang.

## Funding

This work was supported by the National Natural Science Foundation of China (Grant 82574449).

## Disclosure

All authors have read and approved the final version of the manuscript. Qian Li had full access to all of the data in this study and takes complete responsibility for the integrity of the data and the accuracy of the data analysis. Qian Li affirms that this article is an honest, accurate, and transparent account of the study being reported; that no important aspects of the study have been omitted; and that any discrepancies from the study as planned (and, if relevant, registered) have been explained. The funder had no role in the study design; collection, analysis, or interpretation of the data; preparation of the manuscript; or the decision to submit the manuscript for publication.

## Ethics Statement

All animal experiments were conducted in accordance with the institutional guidelines and were approved by the Ethics Committee of China Pharmaceutical University (Approval Number YSL‐202508025). The hucMSCs used in this study were commercially obtained from Jiangsu Puzhongkang Biotechnology Co., Ltd. (Taizhou, Jiangsu, China). According to the supplier, the cells were isolated from voluntarily donated human umbilical cords, and written informed consent for the donation and research use of the tissues was obtained from all donors.

## Conflicts of Interest

The authors declare no conflicts of interest.

## Data Availability

The data that support the findings of this study are available from the corresponding author upon reasonable request.
